# *O*-GlcNAcylation mapping of single living cells by *in situ* quantitative SERS imaging †

**DOI:** 10.1039/d2sc03881a

**Published:** 2022-08-01

**Authors:** Yuanjiao Yang, Yunlong Chen, Shiya Zhao, Huipu Liu, Jingxing Guo, Huangxian Ju

**Affiliations:** State Key Laboratory of Analytical Chemistry for Life Science, School of Chemistry and Chemical Engineering, Nanjing University Nanjing 210023 China hxju@nju.edu.cn; Department of Medical Imaging, Jinling Hospital, School of Medicine, Nanjing University Nanjing 210002 China

## Abstract

*O*-GlcNAcylation is involved in many biological processes including cancerization. Nevertheless, its *in situ* quantification in single living cells is still a bottleneck. Here we develop a quantitative SERS imaging strategy for mapping the *O*-GlcNAcylation distribution of single living cells. *O*-GlcNAcylated compounds (OGCs) can be quantified through their *in situ* azide labeling and then a click reaction competing with azide and Raman reporter labeled 15 nm-gold nanoparticles (AuNPs) for linking to dibenzocyclooctyne labeled 40 nm-AuNPs to produce OGC-negatively correlated SERS signals. The calibration curve obtained *in vitro* can be conveniently used for detecting OGCs in different areas of single living cells due to the negligible effect of cell medium on the click linkage and Raman signal. This method has been successfully applied in mapping *O*-GlcNAcylation distribution in different cell lines and monitoring *O*-GlcNAcylation variation during cell cycling, which demonstrate its great practicability and expansibility in glycosylation related analysis.

## Abstract 2

A quantitative SERS imaging strategy is developed for *O*-GlcNAcylation mapping of single living cells through a competitive click reaction.

## Graphical abstract

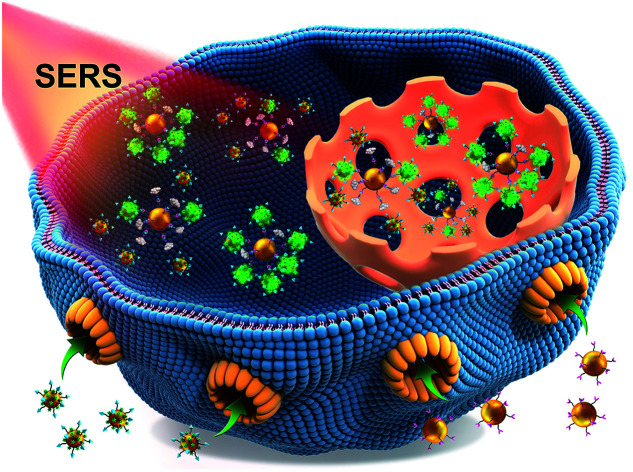

## Introduction

*O*-GlcNAcylation of proteins is a vital protein post-translational modification, which links single *N*-acetylglucosamine (GlcNAc) residues to O-linkages (serine or threonine) on proteins.^1–4^ More than one thousand proteins can be *O*-GlcNAcylated. Thus *O*-GlcNAcylated proteins are abundant in the nucleus, cytosol and other cellular compartments, and *O*-GlcNAcylation plays key roles in the regulation of different cellular processes, including transcription, translation, protein trafficking, and degradation, and has cross talk with phosphorylation of proteins.^5,6^ An abnormal level of *O*-GlcNAcylation causes numerous chronic diseases such as diabetes, Alzheimer's disease, and cancer. Especially, *O*-GlcNAcylation occurs on most of the oncoproteins and hence regulates cancer cell growth, cycle, adhesion and cancer progression.^5,6^ Thus, the analysis of *O*-GlcNAcylation in cancer cells is of great importance for revealing *O*-GlcNAcylation related biological processes and cancer etiology.

Mass spectroscopy is a general technology used to detect *O*-GlcNAcylation.^7^ However, the extreme lability of *O*-GlcNAc in ionization limits its application in *O*-GlcNAcylation analysis.^6,7^ A chemoenzymatic method has been developed for gel electrophoresis analysis of *O*-GlcNAcylated proteins through biotin-streptavidin recognition,^8^ and some fluorescence imaging strategies have been proposed for the visualization of *O*-GlcNAcylation in living cells.^4,9^ For example, Wittmann *et al.* employed green fluorescent protein and metabolic engineering to respectively label the target protein and *O*-GlcNAc for the visualization of protein-specific *O*-GlcNAcylation,^4^ and our previous work designed succinylated wheat germ agglutinin and GlcNAc functionalized gold nanoparticles (AuNPs) as a dual-color fluorescent probe to simultaneously visualize *O*-GlcNAcylated proteins and dissociated GlcNAc residues within living cells.^9^ Obviously, these methods cannot give quantitative results. The *in situ* quantification of *O*-GlcNAcylation inside single living cells is still a bottleneck.

**Scheme 1:**
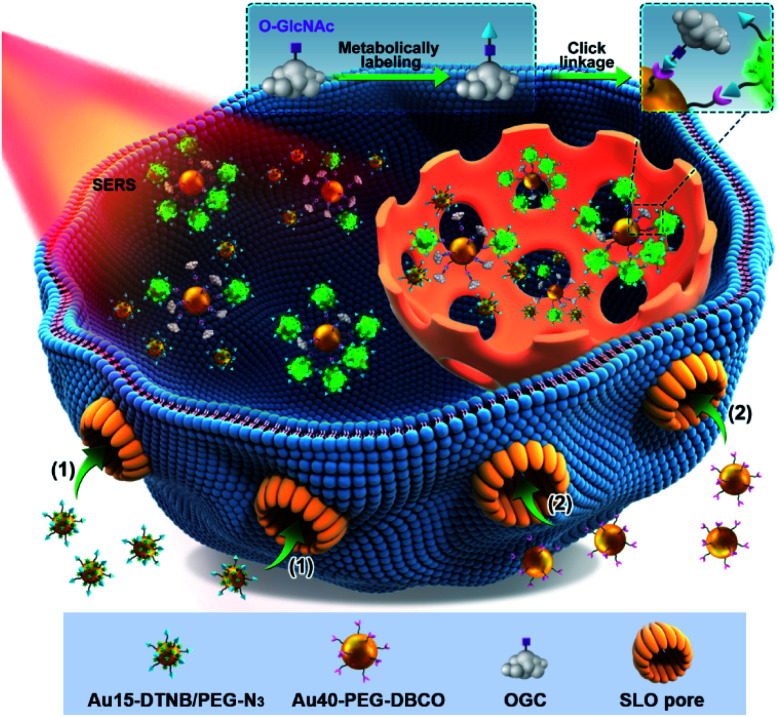
Schematic illustration of the quantitative SERS imaging strategy for *O*-GlcNAcylation mapping of signal living cells. After metabolic labeling and perforation by a pore-forming protein (SLO), Au15-DTNB/PEG-N_3_ and Au40-PEG-DBCO enter the cells in sequence and then a competitive click reaction with azide labeled *O*-GlcNAcylated compounds (OGCs) occurs, which form different aggregates due to different distributions of OGCs in single living cells.

Surface-enhanced Raman spectroscopy (SERS) is a highly sensitive analytical technique and can provide stable and inherent signals of report molecules without interference from complex biological systems.^10,11^ It has been used to detect the glycan^12,13^ and even protein-specific glycan^14^ on the living cell surface. To break through the bottleneck in *in situ* quantification of intracellular *O*-GlcNAcylation, here we develop a quantitative SERS imaging strategy to display for the first time the regional distribution of *O*-GlcNAcylation. This strategy can map the *O*-GlcNAcylation of single living cells through metabolic labeling of *O*-GlcNAcylated compounds (OGCs) with azide^15–18^ and then the competitive click reaction^19^ of dibenzocyclooctyne (DBCO) labeled 40 nm-AuNPs (Au40-PEG-DBCO) with azide labeled OGC and Au15-DTNB/PEG-N_3_, azide 15 nm-AuNPs modified with Raman reporter 5,5-dithiobis (2-nitrobenzoic acid) (DTNB) (Scheme 1). To avoid the effects of probe endocytosis and cellular vesicles on the click reaction, the cells were treated with a pore-forming protein, streptolysin O (SLO), to form pores of over 100 nm on the cell membrane,^20^ which leads to direct probe delivery and can maintain the cell activity.^21^ Using a calibration curve obtained *in vitro*, the concentrations of OGC in different areas of single living cells can be detected for *O*-GlcNAcylation mapping. The successful mapping of GlcNAcylation distributions in different target cells demonstrated its practicability and expansibility.

## Results and discussion

### Copper-free click linkage between Au15-DTNB/PEG-N_3_ and Au40-PEG-DBCO

**Fig. 1:**
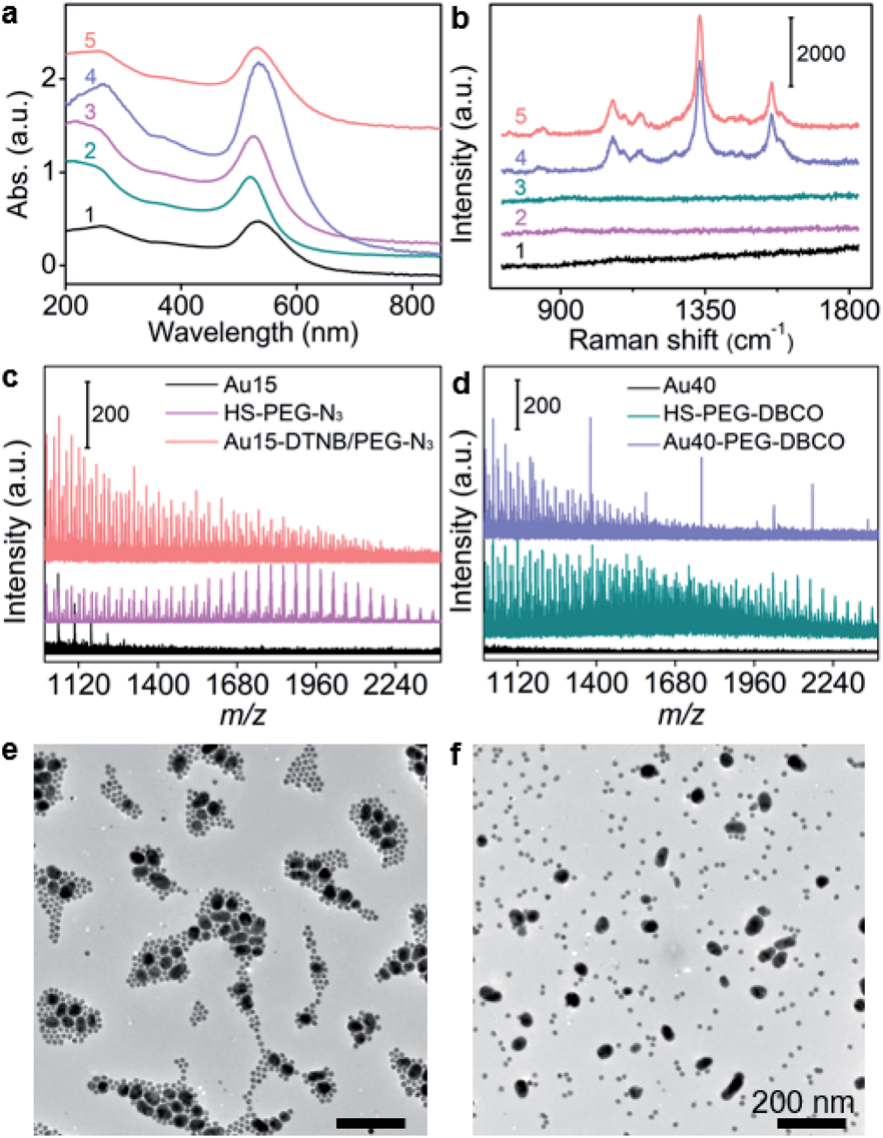
(a) UV-vis absorption spectra of Au40 (1), Au15 (2), Au15-DTNB/PEG-N_3_ (3), and Au40-PEG-DBCO (4) and click aggregates of Au15-DTNB/PEG-N_3_ and Au40-PEG-DBCO (5). (b) Raman spectra of Au15-DTNB/PEG-N_3_ (1), Au40-PEG-DBCO (2), and the mixture of Au15-DTNB/PEG-N_3_ and Au40-PEG-N_3_ as the control (3), and click aggregates of Au15-DTNB/PEG-N_3_ and Au40-PEG-DBCO in 1640 medium (4) or water (5). (c and d) MALDI-TOF mass spectra of Au15-DTNB/PEG-N_3_ (c) and Au40-PEG-DBCO (d). (e and f) TEM images of click aggregates of Au15-DTNB/PEG-N_3_ and Au40-PEG-DBCO (e) and the mixture of Au15-DTNB/PEG-N_3_ and Au40-PEG-N_3_ (f) in 1640 medium. Scale bars: 200 nm.

15 nm-AuNPs and 40 nm-AuNPs were synthesized^22^ for the preparation of Au15-DTNB/PEG-N_3_ and Au40-PEG-DBCO and characterized with transmission electron microscopy (TEM) images and UV-Vis spectra. Au15-DTNB/PEG-N_3_ and Au40-PEG-DBCO retained the original morphology of AuNPs (Fig. S1, S2a and b†), but exhibited a slight red shift of the absorption peak compared to Au15 and Au40 (Fig. 1a), respectively, indicating a slight size increase upon the modification. The individual Au15-DTNB/PEG-N_3_ did not show the Raman characteristic peaks of DTNB (Fig. 1b) due to the negligible SERS effect of single Au15.^14,23^ The MALDI-TOF mass spectra of both Au15-DTNB/PEG-N_3_ and Au40-PEG-DBCO (Fig. 1c and d) exhibited equally spaced *m*/*z* values of 44 corresponding to the ethylene glycol units in PEG, which indicated the successful PEG modification of Au15 and Au40.

The copper-free click reaction between Au15-DTNB/PEG-N_3_ and Au40-PEG-DBCO was firstly validated with TEM images, which showed obvious aggregates (Fig. S2c†) and indicated the successful click linkage of Au15 and Au40 in water. The linkage led to a wider absorption peak than individual Au15-DTNB/PEG-N_3_ or Au40-PEG-DBCO (Fig. 1a) and the characteristic peaks of DTNB at 1332 cm^−1^ and 1580 cm^−1^ (Fig. 1b), proving the SERS effect of Au40 to DTNB. Moreover, the click aggregates obtained in 1640 medium showed the same Raman spectrum as those obtained in water (Fig. 1b, curves 4 and 5), indicating the negligible effect of cell medium on the click linkage and Raman signal. Besides, the click linkage also occurred in RPMI-1640 medium (Fig. 1b and e). In contrast, the mixture of Au15-DTNB/PEG-N_3_ and Au40-PEG-N_3_ did not show the characteristic.

The Raman peaks of DTNB (Fig. 1b) or obvious aggregates (Fig. 1f) demonstrate the good stability of these probes and exclude their aggregation in the absence of click linkage.

### Calibration curve for OGC quantification

**Fig. 2:**
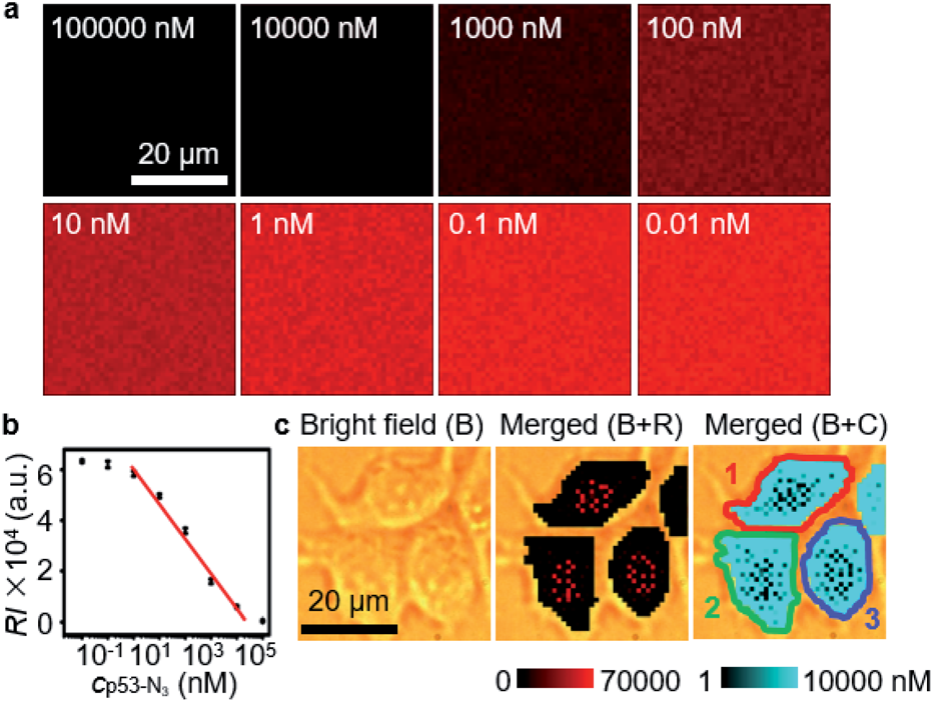
(a) SERS imaging of click aggregates of Au15-DTNB/PEG-N_3_ and Au40-PEG-DBCO in 1640 medium containing different concentrations of p53-N_3_. (b) Calibration curve for OGC quantification. (c) SERS imaging (R) and *O*-GlcNAcylation mapping (C) of MCF-7 cells.

The quantification ability of the designed competitive strategy was verified *in vitro* using human p53 (ref. 24–27) as the model glycoprotein, which is *O*-GlcNAcylated at serine 149.^24,28^ The p53 and azide-labeled p53 (p53-N_3_) were immunocaptured from MCF-7 cells and GlcNAz treated MCF-7 cells, respectively, which exhibited a slight *m*/*z* difference in mass spectra (Fig. S3a†) and tiny band movement in SDS-PAGE analysis (Fig. S3b†), indicating the single N_3_ group on p53-N_3._ The concentration of the obtained p53-N_3_ could be detected to be 10.9 μM through its reaction with excessive DBCO-Cy5 and then recording the fluorescent signal of Cy5 after removing the extra DBCO-Cy5 by ultrafiltration (Fig. S4†). After the mixtures of different concentrations of p53-N_3_ and 1 nM Au15-DTNB/PEG-N_3_ were incubated with 1 nM Au40-PEG-DBCO in RPMI-1640 medium for 1 h, they were subjected to SERS imaging under the same conditions as cell imaging. The SERS signal responding to the aggregates of Au15-DTNB/PEG-N_3_ and Au40-PEG-DBCO increased with decreasing p53-N_3_ concentration (Fig. 2a), demonstrating the presence of competitive binding of Au15-DTNB/PEG-N_3_ and p53-N_3_ with Au40-PEG-DBCO.

The competitive binding aggregates at different p53-N_3_ concentrations (1 and 10 000 nM) could be obviously distinguished in TEM images (Fig. S5†). Besides, the SERS signal of the aggregates in 1 nM (Fig. S6a and c†) and 10 000 nM (Fig. S6b and d†) p53-N_3_ solution remained unchanged from 0.5 h to 8 h, which indicated the stability of the aggregates. The average signal intensity (RI) was inversely proportional to the logarithm value of p53-N_3_ concentration (lg*c*p53-N_3_) ranging from 1 to 10 000 nM (Fig. 2b) with an equation of RI = −13946 lg*c*p53-N_3_ + 61 308 (*R*^2^ = 0.98616). Thus, a quantitative method could be provided for p53-N_3_ analysis and detecting the concentration of p53 or OGCs in single living cells after metabolic labeling with GlcNAz due to the negligible effect of cell medium on the click linkage and Raman signal (Fig. 1b, curves 4 and 5).

### *In situ O*-GlcNAcylation mapping of living cells

To achieve the quantification of metabolically labeled OGCs in living cells, the GlcNAz-treated cells were firstly perforated with SLO for avoiding the effects of probe endocytosis and cellular vesicles on the click reaction. Propidium iodide (PI) staining confirmed the formation of pores on SLO-treated cells (Fig. S7a and b†).^21^ In addition, the pores could remain on the cell membrane for 12 h, which was sufficient for the entry of probes (Fig. S7c†). The conditions for competitive click linkage in metabolically labeled and perforated living cells were optimized in the presence of iodoacetamide (IAM), which could block the undesired thiol to inhibit its reaction with DBCO and improve the cycloaddition efficiency between –N_3_ and DBCO.^29^ The optimal concentrations and incubation times of Au15-DTNB/PEG-N_3_ and Au40-PEG-DBCO were 1 nM for 2 h and 0.8 nM for 4 h (Figs. S8–S11†), respectively. After all these treatments, the cells could retain approximate 90% viability (Fig. S12†), suggesting their acceptable compatibility for living cell analysis.

Under the optimized incubation conditions, obvious SERS signals were distributed in different regions of MCF-7 cells (Fig. 2c, B+R), which could be converted to the concentration of OGCs with the average RI and the calibration curve, and thus led to a concentration mapping of OGCs (Fig. 2c, B+C). By merging the bright field image, the average concentration of OGCs in living single cells could be quantified (Fig. 2c). Thus, the *in situ O*-GlcNAcylation mapping of living MCF-7 cells was for the first time achieved.

**Fig. 3:**
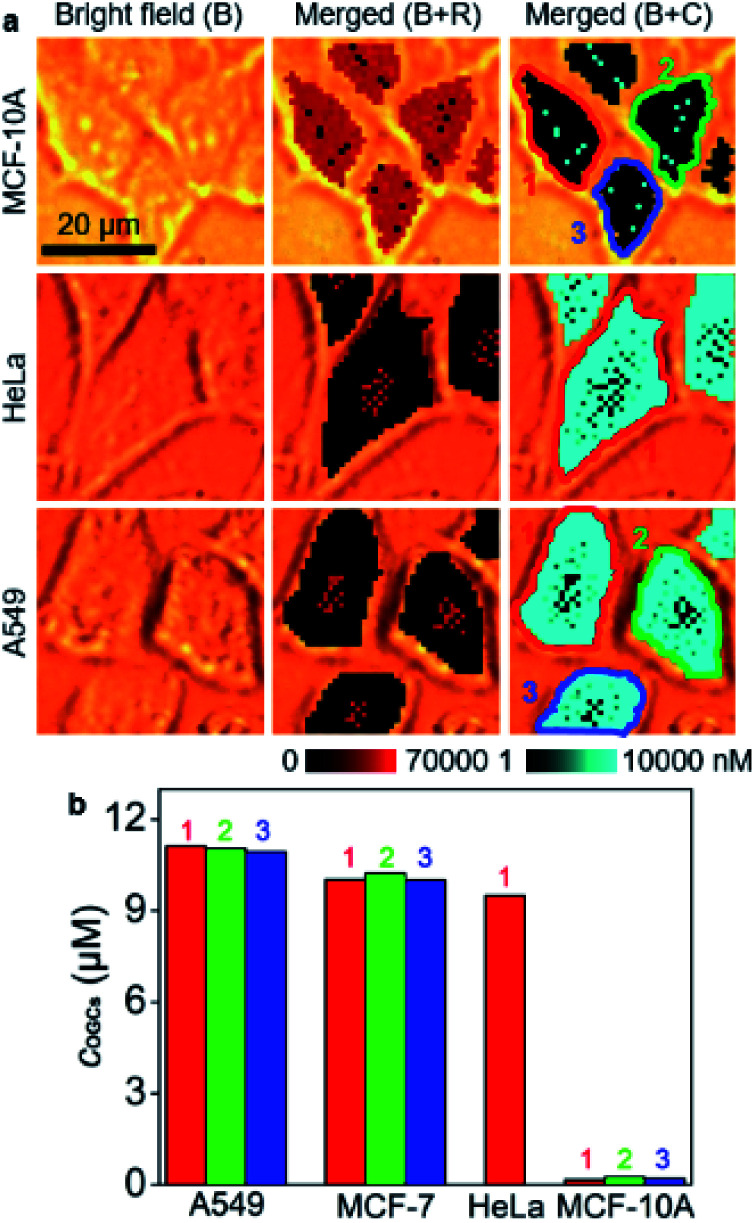
(a) SERS imaging and *O*-GlcNAcylation mapping of MCF-10A, HeLa and A549 cells. (b) Statistical *O*-GlcNAcylation mapping intensities of every single cell in (a) and Fig. 2c.

The developed strategy for *O*-GlcNAcylation mapping was firstly applied to quantitatively evaluate the distribution of *O*-GlcNAcylation in MCF-10A, HeLa and A549 cells (Fig. 3a). Similarly, *O*-GlcNAcylation was unevenly distributed in these cells. The average concentrations of OGCs in single cells were calculated to be 11.0 μM for A549 cells, 10.1 μM for MCF-7 cells, 9.5 μM for HeLa cell, and 0.21 μM for MCF-10A (Fig. 3b), which revealed significantly different *O*-GlcNAcylation levels between tumor and healthy cells. Moreover, confocal fluorescence imaging and flow cytometric analysis qualitatively validated the results. After metabolically labeled and perforated cells were stained with DBCO-Cy5, the cells were subjected to CLSM imaging (Fig. S13†) and flow cytometric assay (Fig. S14†), which showed the same difference of *O*-GlcNAcylation levels among these cells. However, these fluorescence methods provided only the qualitative results and rely on the complete cleanup of the unconjugated dyes inside cells, which brings about false positive results. Therefore, the designed competitive SERS strategy exhibits great reliability and convenience in *in situ* quantitative analysis of intracellular *O*-GlcNAcylated compounds.

The proposed strategy was then used for monitoring of *O*-GlcNAcylation variation upon alloxan treatment, which can down-regulate the *O*-GlcNAcylation level.^9,30^ With increasing alloxan concentration, a stronger SERS signal was observed, indicating a down-regulated *O*-GlcNAcylation level (Fig. S15a†). From the concentration maps, the average concentrations of OGCs in every single cell with different inhibitions could be obtained (Fig. S15b†). The inhibition effect quantitatively agreed with the fluorescence imaging analysis (Fig. S16†).

**Fig. 4:**
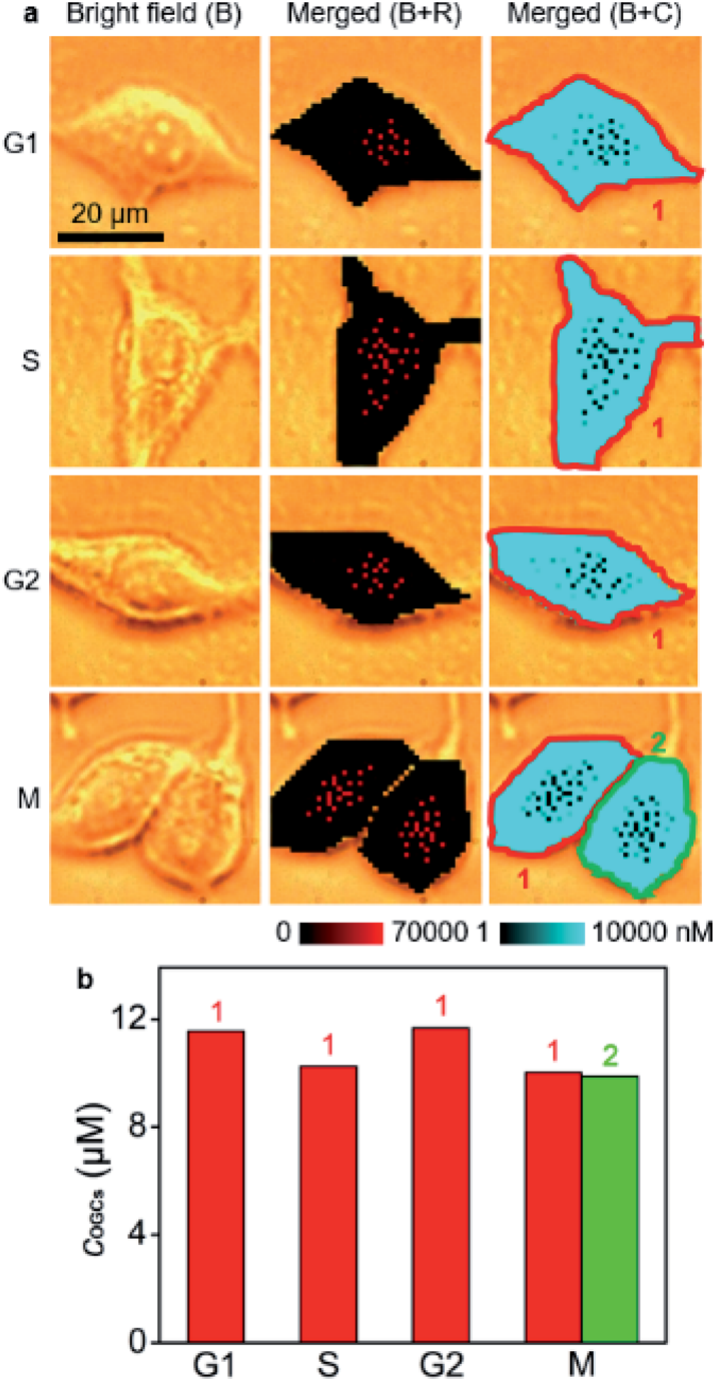
(a) SERS imaging and *O*-GlcNAcylation mapping in MCF-7 cells in G1, S, G2, and M phases. (b) Statistical *O*-GlcNAcylation mapping intensities of every single cell in (a).

*O*-GlcNAcylation plays a crucial role in regulating DNA replication, cell-cycle progression and mitosis, and is related to cancerization.^31–33^ It also protects genome integrity by modifying histones, kinases and scaffold proteins in the face of an altered cell cycle.^34^ Therefore, the quantification of *O*-GlcNAcylation during cell cycling is of great importance. To use the developed strategy for the quantification of the *O*-GlcNAcylation level in different cell phases, MCF-7 cells were synchronized at G1/S using the thymidine double-block method^35,36^ and then incubated in fresh medium for 6 h, 13 h, 23 h, and 24 h to reach S-, G2-, M- and G1-phases, respectively (Fig. S17†). Afterward, the cells were metabolically labeled with GlcNAz for 34 h, which was shorter than a complete cycle of MCF-7 cells^35,36^ for guaranteeing them in the original cell phases (Fig. S18†). Thus, the *O*-GlcNAcylation levels as well as their distributions in different cell phases could be detected with the proposed quantitative SERS imaging method (Fig. 4a). The average concentrations of OGCs in single cells were calculated to be 11.6 μM in the G1 phase, 10.3 μM in the S phase, 11.7 μM in the G2 phase, and 10.0 μM in the M phase of MCF-7 cells (Fig. 4b). The difference of *O*-GlcNAcylation levels in different cell phases could be attributed to the corresponding activity of *O*-GlcNAc transferase^37,38^ and *O*-GlcNAcase.^39^ The *O*-GlcNAcylation variation during the cell cycle was coincident with the intensity change of fluorescence images (Fig. S19†), demonstrating the reliability.

## Conclusions

In conclusion, we develop a quantitative SERS imaging strategy to display for the first time the regional distribution of *O*-GlcNAcylation inside single living cells through metabolic labeling of OGCs with azide for linking to Au40-PEG-DBCO competitively with azide labeled Au15-DTNB/PEG-N_3_, which lead to a signal switch negatively correlated OGC concentration. Combining with the *in vitro* obtained calibration curve with a linear range from 1 to 10 000 nM, the concentrations of OGCs in different areas of a single cell can be directly quantified. The developed strategy has successfully been used for the detection of *O*-GlcNAcylation levels in MCF-7, MCF-10A, A549, and HeLa cells, and the monitoring of *O*-GlcNAcylation variation responding to its inhibitor and cell cycle. The designed cell imaging procedure, including metabolic labelling, SLO perforation, probe delivery and intracellular competitive click linkage, can maintain the viability of target cells. This strategy can be conveniently expanded to quantitatively map other –N_3_ labelled biomolecules in living cells, and provides a practical tool for the exploration of *O*-GlcNAcylation related biological mechanisms.

## Data availability

All relevant data is presented in the paper and ESI.† Raw data is available upon request by email to the corresponding author.

## Author contributions

Y. Y., Y. C., and H.J. designed the projects, planned the experiments and wrote the manuscript; Y. Y. performed Raman and cell related experiments. Y. C. carried out fluorescence experiments. S. Z. prepared and characterized the AuNP probe. H. L. and J. G. cultured cells and analyzed data. H. J. supervised and coordinated all investigators for this project.

## Conflicts of interest

There are no conflicts to declare.

## Supplementary Material

SC-013-D2SC03881A-s001
