# Supplementary material for: O-GlcNAcylation mapping of single living cells by in situ quantitative SERS imaging
Source: Chem Sci. 2022 Aug 1;13(33):9701–5. doi: 10.1039/d2sc03881a (PMC9400686; doi:10.1039/d2sc03881a)
Supplement: SC-013-D2SC03881A-s001 [file SC-013-D2SC03881A-s001.pdf]

Electronic Supplementary Material (ESI) for Chemical Science.
This journal is © The Royal Society of Chemistry 2022

Supplementary Information

O-GlcNAcylation Mapping of Single Living Cells by In-situ Quantitative SERS Imaging

Yuanjiao Yang, [†a] Yunlong Chen, [†a] Shiya Zhao, [a] Huipu Liu, [a] Jingxing Guo [b] and Huangxian Ju [*a]

[a]State Key Laboratory of Analytical Chemistry for Life Science, School of Chemistry and Chemical Engineering, Nanjing University, Nanjing 210023, China.

[b]Department of Medical Imaging, Jinling Hospital, School of Medicine, Nanjing University, Nanjing 210002, China.

[†]These authors contributed equally to this work.

*e-mail: hxju@nju.edu.cn

Supplementary materials and apparatus

Materials. Chloroauric acid ($HAuCl_4 \cdot 3H_2O$, 99%), trisodium citrate, streptolysin O (SLO), 5,5'-dithiobis(2-nitrobenzoic acid) (DTNB), alloxan, thymidine, dibenzocyclooctyne-Cy5 (DBCO-Cy5), iodoacetamide (IAM), protease inhibitor, and dimethyl sulfoxide (DMSO) were purchased from Sigma-Aldrich Inc. (USA). MCF-7, A549, HeLa, MCF-10A cells, RPMI-1640 cell culture medium, Dulbecco's Modified Eagle's Medium (DMEM), trypsin, phosphate buffered saline (PBS) (pH 7.4, containing 136.7 mM NaCl, 2.7 mM KCl, 8.72 mM Na_2HPO_4, 1.41 mM KH_2PO_4, 1 mM $CaCl_2$, and 1 mM $MgCl_2$), and Hank's balanced salt solution (HBSS) were supplied by KeyGen Biotech. Co. Ltd. (China). Click-iT® tetraacetylated N-azidoacetylglucosamine (GlcNAz), fetal bovine serum (FBS), PageRuler™ prestained protein ladder (10 to 180 kDa), and Alexa Fluor™ 647 NHS Ester (AF647-NHS) were from Thermo Fisher Scientific Inc. (USA). P53 immunocapture kit was obtained from Abcam PLC Co., Ltd. (USA). RIPA lysis buffer I, and phenylmethanesulfonyl fluoride (PMSF) were obtained from Shanghai Sangon Biotech. Co., Ltd. (China). Azide polyethylene glycol 2000 thiol (HS-PEG-N_3) and dibenzocyclooctyne polyethylene glycol 2000 thiol (HS-PEG-DBCO) were provided by Shanghai Toyongbio Tech. Inc. (China). All aqueous solutions were prepared using ultrapure water (≥18 MΩ, Milli-Q, Millipore).

Apparatus. Transmission electron microscopic (TEM) images were obtained on a JEM-2100 transmission electron microscope (JEOL Ltd., Japan). Confocal fluorescence micrographs were acquired on a TCS SP8 confocal laser scanning microscope (CLSM) (Leica, Germany). The UV-vis absorption spectra were recorded using a UV-vis spectrophotometer (Nanodrop-2000C, Nanodrop, USA). Flow cytometric analysis was gained on a Coulter FC-500 flow cytometer (Beckman-Coulter). Sodium dodecyl sulfate-polyacrylamide gel electrophoresis (SDS-PAGE) analysis was performed on an Electrophoresis Analyzer (Thermo Fisher Scientific, USA) and imaged on a Bio-Rad ChemDoc XRS (Bio-Rad, USA). MALDI-TOF mass spectra were recorded on 4800 plus MALDI TOF/TOF analyzer (AB sciex, U.S.A.) with a Nd:YAG laser at 355 nm. BCA protein quantification and CCK8 assays were performed on a Varioskan Flash spectral scanning multimode reader (Thermo Fisher Scientific, USA). Raman spectra and images were recorded on a Renishaw inVia confocal Raman microscope (Renishaw, UK) using a 50-times telephoto objective under 633 nm laser excitation with exposure time of 1s.

Synthesis of AuNPs. Gold nanoparticles (AuNPs) was synthesized following previous method.[1] 15-nm and 40-nm AuNPs (Au15 and Au40) were prepared by quickly adding 1 mL and 0.5 mL trisodium citrate (1% wt) to 50 mL boiling $HAuCl_4$ solution (0.01% wt). The mixtures were kept boiling until the color became red, and remained for 10 min before cooling to room temperature. The obtained AuNPs were washed by centrifugation under 11600 g for Au15 and 3500 g for Au40. The concentrations of the prepared AuNPs were estimated by UV-vis absorption spectrometry.[2]

Preparation of Au15-DTNB/PEG-N_3 and Au40-PEG-DBCO. 10 µL DTNB (10 mM in ethanol) and 10 µL HS-PEG-N_3 (10 mM) were added to 1 mL Au15 (10 nM), and shaken at room temperature overnight. Similarly, 10 µL HS-PEG-DBCO (10 mM) was added to 1 mL Au40 (10 nM), and shaken at room temperature overnight. Then the products were washed by centrifugation under 11600 g for Au15 and 3500 g for Au40 twice to obtain Au15-DTNB/PEG-N_3 and Au40-PEG-DBCO, respectively, which were resuspended in 500 µL water.

Cell culturing. MCF-7, MCF-10A and A549 cells were separately cultured in RPMI-1640 medium supplemented with 10% fetal bovine serum (FBS), penicilin (0.1 mg/mL), and streptomycin (0.1 mg/mL). HeLa cells were cultured in DMEM supplemented with 10% FBS, streptomycin (0.1 mg/mL), and penicillin (0.1 mg/mL). All cells were grown at 37 °C in a humidified chamber containing 5% CO_2.

Perforation and metabolic labeling of cells. The perforation of cells with SLO was followed to the previously reported protocol.[3,4] SLO was firstly activated with 5 mM dithiothreitol at 37 °C for 2 h, and then stored in small aliquots at -20 °C. Cells seeded on confocal plates were cultured overnight, and then washed with HBSS for three times. After incubated with GlcNAz (40 µM) for 48 h, the cells were perforated with 100 U mL^{-1} SLO in HBSS at 37 °C for 30 min. The cell perforation was verified by incubating the perforated cells

with PI (10 μg mL^{-1}) in 1% BSA-containing HBSS at 37 °C for 20 min. After gently washing with PBS for three times, the cells were imaged by CLSM under excitation at 535 nm. The fluorescence emission was collected from 555 nm to 625 nm.

Extraction of p53 or p53-N_3 from cells. The cells were lysed with cold RIPA lysis buffer I containing protease inhibitor and PMSF. The lysate was transferred to a centrifuge tube and shaken for 30 min at 4 °C to collect the supernatant by centrifugation at 16000 g for 20 min at 4 °C, which was subjected to p53 purification using p53 Immunocapture kit according to the manufacturer's procedure. p53-N_3 from GlcNAz metabolically labeled cells was obtain with the same procedure, which contained in fact low proportion of p53 considering the labelling efficiency. The obtained p53 or p53-N_3 was characterized with SDS-PAGE analysis and mass spectrometry, and their concentrations were detected with BCA protein assay kit.

SDS-PAGE analysis. The p53 and p53-N_3 obtained above were mixed with LDS sample buffer and heated at 70 °C for 10 min. The mixtures were then electrophoresed on 4-12% bis-tris protein gels (1.5 mm, 15-well) in MOPS SDS running buffer (1×) at 200 V for 50 min. The gels were immersed in Coomassie brilliant blue R-250 with 30-min shaking for staining, followed by destaining with eluent, and finally visualized using a Bio-Red ChemDoc XRS imaging system.

Quantification of obtained p53-N_3. The p53-N_3 obtained above was incubated with 1 mM DBCO-Cy5 at 37 °C for 1 h, which was then ultrafiltrated to remove the unreacted DBCO-Cy5 and obtain p53-DBCO-Cy5. The concentration of the obtained p53-N_3 could thus be determined with the fluorescence intensity (FI) of Cy5.

Calibration curve for quantitative SERS imaging of OGCs. The calibration curve for quantitative SERS imaging of O-GlcNAcylated OGCs was obtained using p53 as a model OGC. The extracted p53-N_3 was firstly diluted in RPMI-1640 medium to different concentrations ranging from 100000 nM to 0.01 nM. 10 μL Au40-PEG-DBCO (100 nM) was added to the mixture of 10 μL Au15-DTNB/PEG-N_3 (100 nM) and 980 μL p53-N_3 solution and incubated at 37 °C for 1 h.

To investigate the stability of SERS signals of the aggregates, 10 μL Au40-PEG-DBCO (100 nM) was added to the mixture of 10 μL Au15-DTNB/PEG-N_3 (100 nM) and 980 μL 10000 nM or 1 nM p53-N_3 solution and incubated at 37 °C for 0.5 h to 8 h.

The mixture was then added into confocal plate to perform the SERS imaging in signal-to-baseline map review mode. The DTNB peak intensity at 1332 cm^{-1} was collected from the points (1 μm × 1 μm) in an area of 40 μm × 40 μm with 633 nm excitation at a laser power of 5.2 mW/μm^2 and an exposure time of 1 s to obtain the average Raman intensity.

Conditions optimization for SERS imaging. After metabolically labeled with GlcNAz and perforated with SLO, the MCF-7 cells were firstly incubated with 0.25-1.25 nM Au15-DTNB/PEG-N_3 for 30-150 min and 1 mM IAM for another 30 min. After washed with RPMI-1640 medium for three times, the cells were incubated with 0.20-1.00 nM Au40-PEG-DBCO for 1-5 h and then washed with RPMI-1640 medium for three times again. The obtained cells were subjected to SERS imaging in 10% FBS-containing RPMI-1640 by collecting the signals at the points (2 μm × 2 μm) in an area of 40 μm × 40 μm. The statistic signal intensity in cells was directly read out through the average of all pixels from Raman images using WiRE 3.4 and Origin 2015 software. The cell viability was analyzed through CCK8 assays according to the instruction.

Quantitative distribution of O-GlcNAcylation in living cells. The Raman images of MCF-7 cells were collected for quantification of O-GlcNAcylation by treating the metabolically labeled and perforated cells with 1 nM Au15-DTNB/PEG-N_3 for 2 h, 0.8 nM Au40-PEG-DBCO for 4 h.

The concentration mapping in the cell area was displayed by converting the Raman intensities of Raman images to concentrations with the calibration curve. The concentrations of OGCs were directly read out through the average of all points inside corresponding cells from concentration images using Origin 2015 software.

To inhibit O-GlcNAcylation, MCF-7 cells were incubated with different concentration of alloxan (1, 5, 10 mM) for 48 h. The treated cells were then metabolically labeled, perforated with SLO, and incubated with Au15-DTNB/PEG-N_3 and Au40-PEG-DBCO under the optimal conditions for SERS imaging. The obtained cells were subjected to SERS imaging in 10% FBS-containing RPMI-1640 by collecting the signals at the

points (1 μm × 1 μm) in an area of 40 μm × 40 μm. The SERS mapping modes in living cells was consistent with that in in vitro calibration experiment. The quantification of O-GlcNAcylation distribution in HeLa, A549, MCF-10A and synchronized MCF-7 cells were performed with the same procedures.

Cell synchronization and flow cytometric analysis. MCF-7 cells were synchronized at the G1/S boundary by incubation with 2 mM thymidine in 10% FBS-containing RPMI-1640 medium for 24 h, followed with a fresh medium for 12 h and thymidine-contained medium for another 24 h. Afterwards, the cells at the G1/S boundary were incubated in fresh medium for another 6 h, 13 h, 23 h, and 24 h to reach S-, G2-, M- and G1-phases, respectively.

After synchronized at different phases, the MCF-7 cells were digested with trypsin and centrifuged at 100 g for 5 min. The cell pellet was fixed in 1 mL 75% ethanol for 30 min, washed with PBS and incubated with 100 μL RNase A (KeyGen Biotech) at 37 °C for 30 min. The obtained cells were stained with propidium iodide (10 μg mL^{-1}) solution at 4 °C for 30 min and dispersed in PBS for flow cytometric analysis of the DNA contents in different cell cycles.

The DNA contents in GlcNAz metabolically labelled and SLO perforated cells at different cycles were validated by flow cytometric analysis with the same procedure.

CLSM imaging of O-GlcNAcylation in living cells. After MCF-7 cells, HeLa, A549, MCF-10A, and synchronized or alloxan-treated MCF-7 cells were metabolically labelled with GlcNAz and perforated with SLO, the cells were incubated with 1 mM IAM for 30 min and stained with DBCO-Cy5 (10 μg mL^{-1}) at 4 ^{o}C for 30 min. The cells without metabolically labelling were used as negative controls. After the cells were carefully washed with PBS for 3 times, they were subjected to CLSM imaging under 631 nm excitation. The fluorescence intensities of these cells were acquired from 650 nm to 750 nm as the average of pixel readouts with Leica Application Suite X.

Supplementary figures

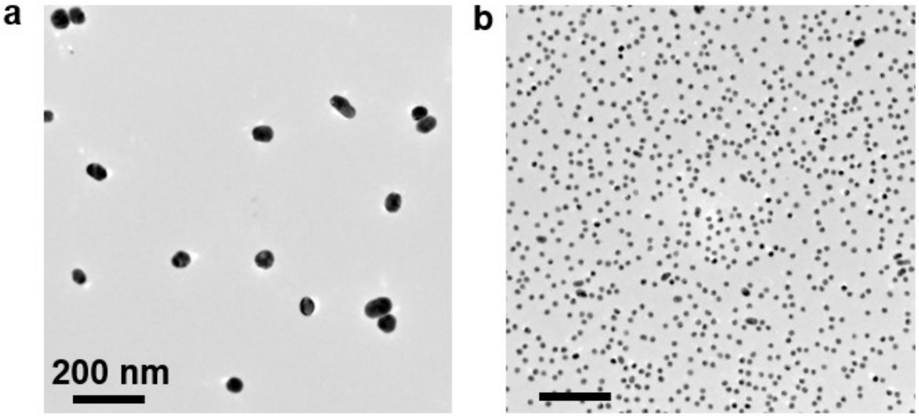

Figure S1 TEM images of Au40 (**a**) and Au15 (**b**). Scale bars: 200 nm.

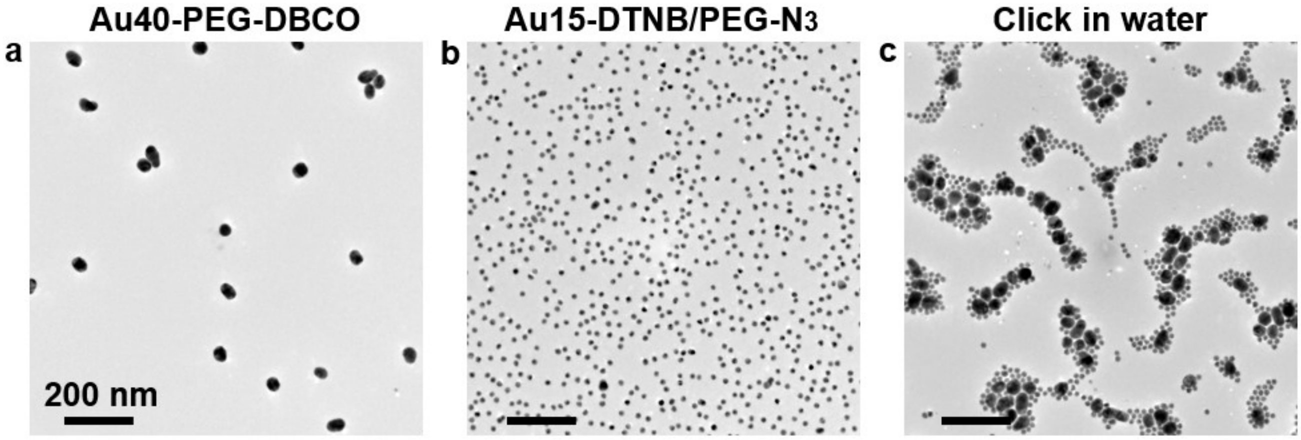

Figure S2 TEM images of Au40-PEG-DBCO (**a**), Au15-DTNB/PEG-N_3 (**b**) and click aggregates of Au40-PEG-DBCO and Au15-DTNB/PEG-N_3 (**c**). Scale bars: 200 nm.

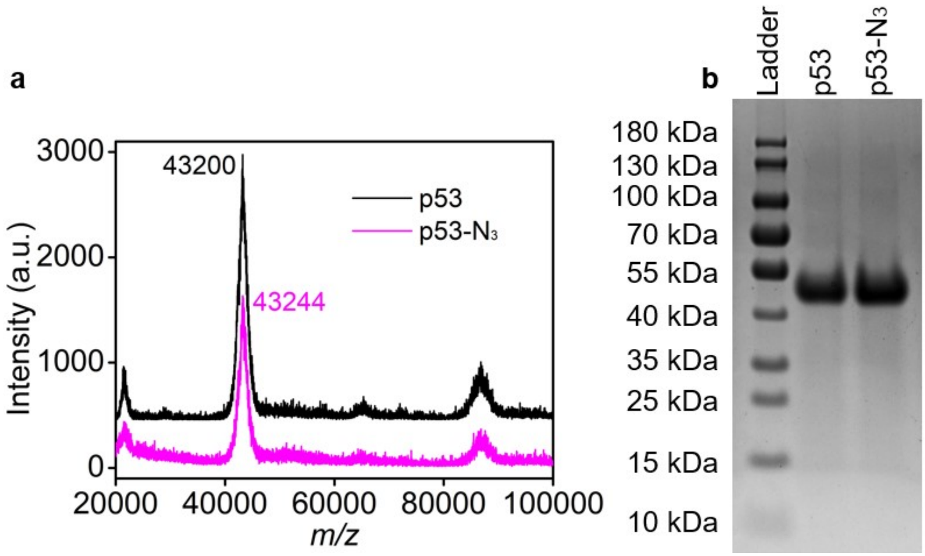

Figure S3 a MALDI-TOF mass spectra of p53-N_3 and p53 immunocaptured from MCF-7 cells after and before metabolic labeling. **b** SDS-PAGE analysis of p53 and p53-N_3 extracted from these cells.

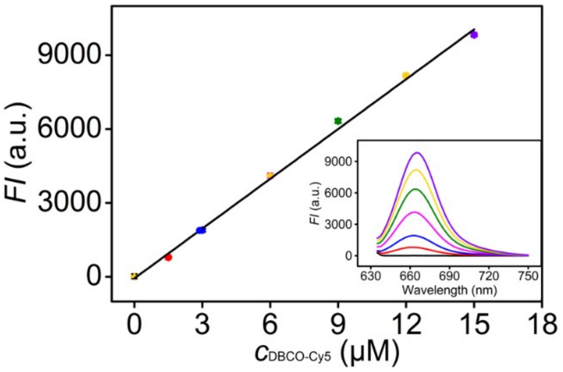

Figure S4 Fluorescent calibration curve of DBCO-Cy5 at fluorescence emission of 665 nm. Inset: fluorescence spectra of DBCO-Cy5 at 0, 1.5, 3, 6, 9, 12 and 15 μM (from bottom to top).

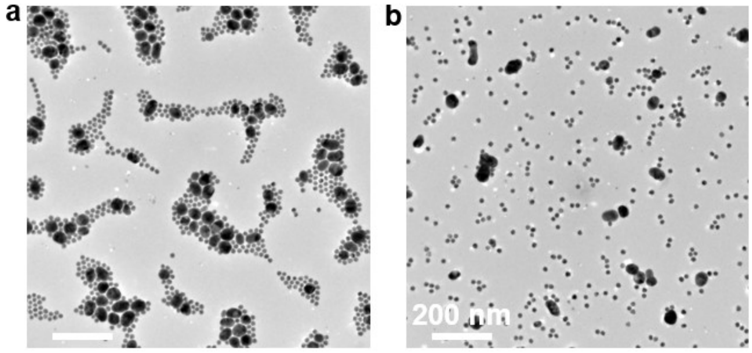

Figure S5 TEM images of click aggregates of Au40-PEG-DBCO, Au15-DTNB/PEG-N_3 and 1 nM (**a**) or 10000 nM p53-N_3 (**b**) in 1640 medium. Scale bars: 200 nm.

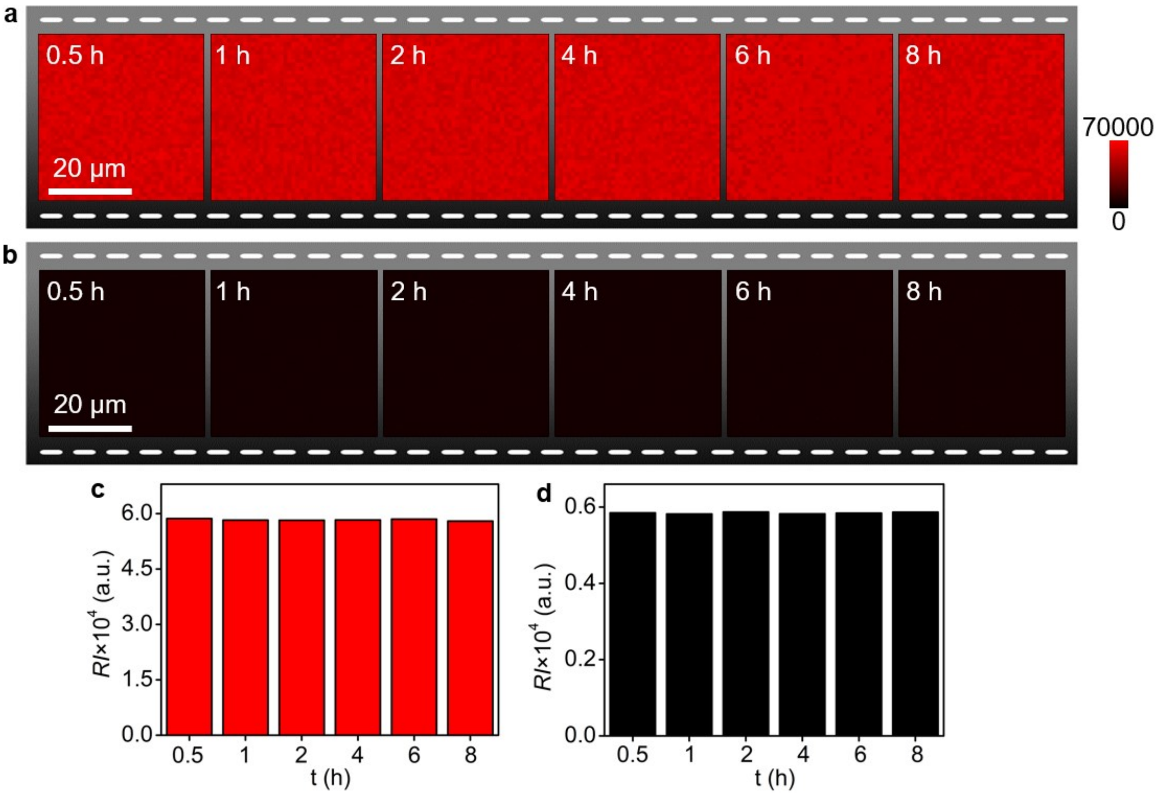

Figure S6 SERS imaging of click aggregates of Au15-DTNB/PEG-N_3 and Au40-PEG-DBCO in 1 nM (**a**) and 10000 nM p53-N_3 solution (**b**) from 0.5 h to 8 h. (**c**) and (**d**) Statistical SERS mapping intensities of every different time in (**a**) and (**b**).

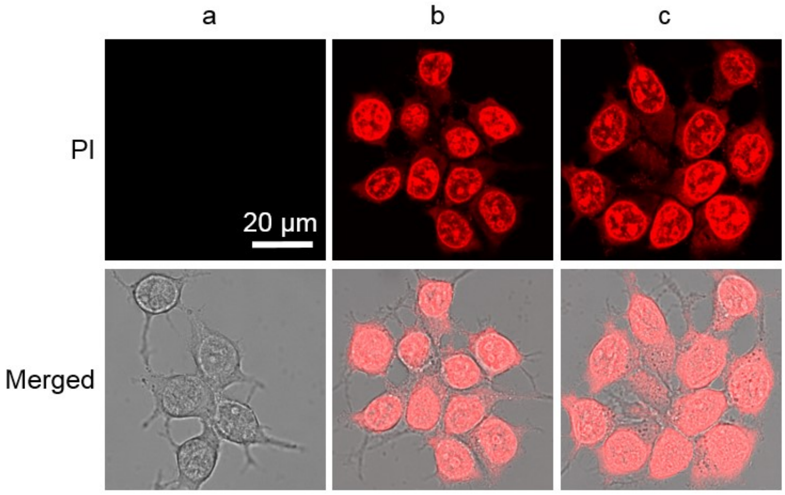

Figure S7 CLSM images of MCF-7 cells (a), SLO-perforated MCF-7 cells (b), SLO-perforated MCF-7 cells in RPMI-1640 medium for 12 h (c), then stained with propidium iodide (PI). Scale bar: 20 μm.

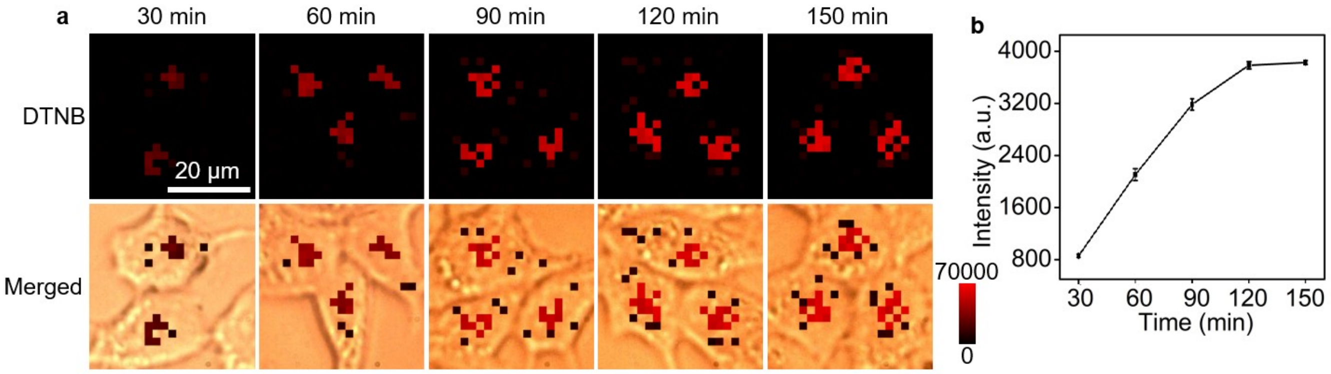

Figure S8 Optimization of incubation time of Au15-DTNB/PEG-N_3. **a** Raman images of metabolically labeled and perforated MCF-7 cells after incubation with 1 nM Au15-DTNB/PEG-N_3 for different times and 1 nM Au40-PEG-DBCO for another 2 h. **b** Plot of statistic Raman intensity from (**a**) vs incubation time. The data error bars indicate mean ± SD (n = 3).

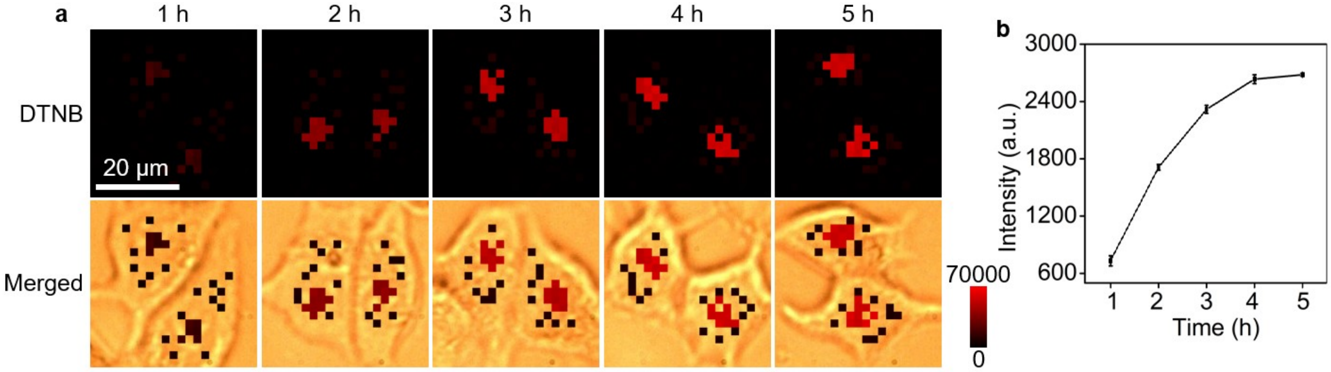

Figure S9 Optimization of incubation time of Au40-PEG-DBCO. **a** Raman images of metabolically labeled and perforated MCF-7 cells after incubation with 1 nM Au15-DTNB/PEG-N_3 for 2 h and then 1 nM Au40-PEG-DBCO for different times. **b** Plot of statistic Raman intensity from (**a**) vs incubation time. The data error bars indicate mean ± SD (n = 3).

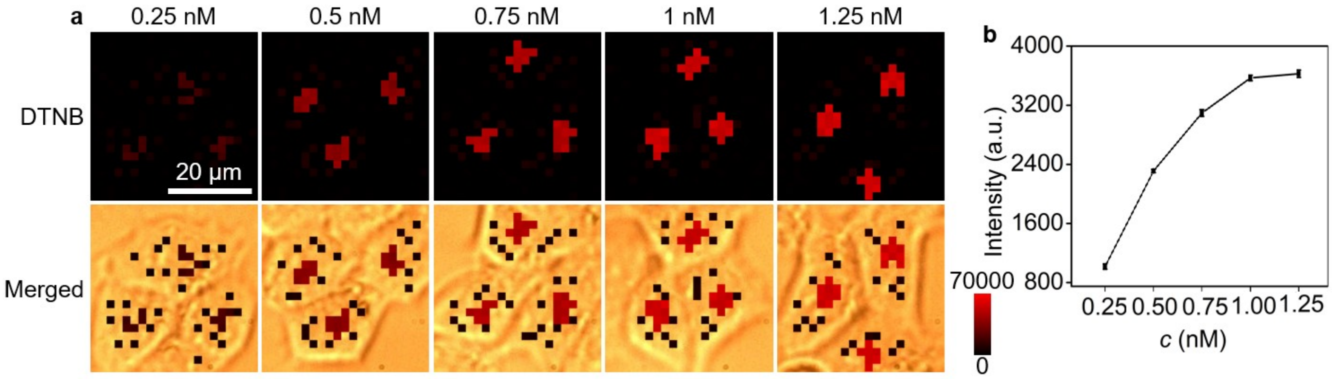

Figure S10 Optimization of incubation concentration of Au15-DTNB/PEG-N_3. **a** Raman images of metabolically labeled and perforated MCF-7 cells after incubation with Au15-DTNB/PEG-N_3 at different concentrations for 2 h and then 1 nM Au40-PEG-DBCO for another 4 h. **b** Plot of statistic Raman intensity from (**a**) vs Au15-DTNB/PEG-N_3 concentration. The data error bars indicate mean ± SD (n = 3).

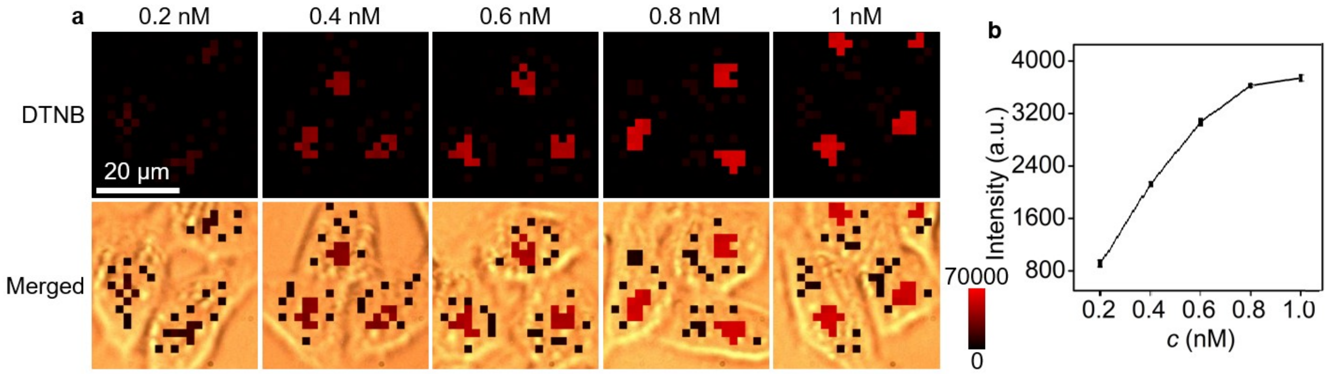

Figure S11 Optimization of incubation concentration of Au40-PEG-DBCO. **a** Raman images of metabolically labeled and perforated MCF-7 cells after incubation with 1 nM Au15-DTNB/PEG-N_3 for 2 h and then Au40-PEG-DBCO at different concentrations for 4 h. **b** Plot of statistic Raman intensity from (**a**) vs Au40-PEG-DBCO concentration. The data error bars indicate mean ± SD (n = 3).

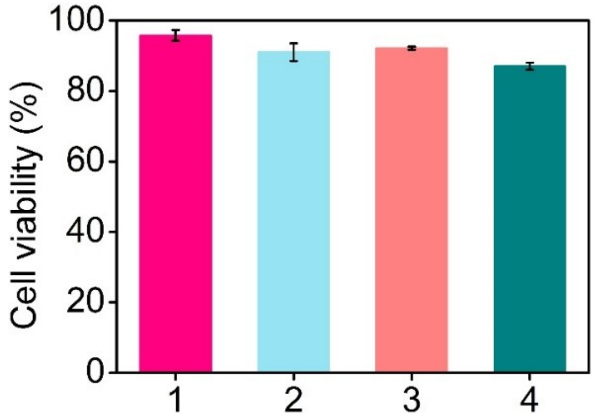

Figure S12 Cell viability of metabolically labeled MCF-7 cells after incubation with SLO (1), SLO and Au15-DTNB/PEG-N_3 (2), SLO, and Au40-PEG-DBCO (3), and SLO, Au15-DTNB/PEG-N_3, IAM and Au40-PEG-DBCO (4) under optimal conditions using CCK8 assay. The data error bars indicate mean ± SD (n = 5).

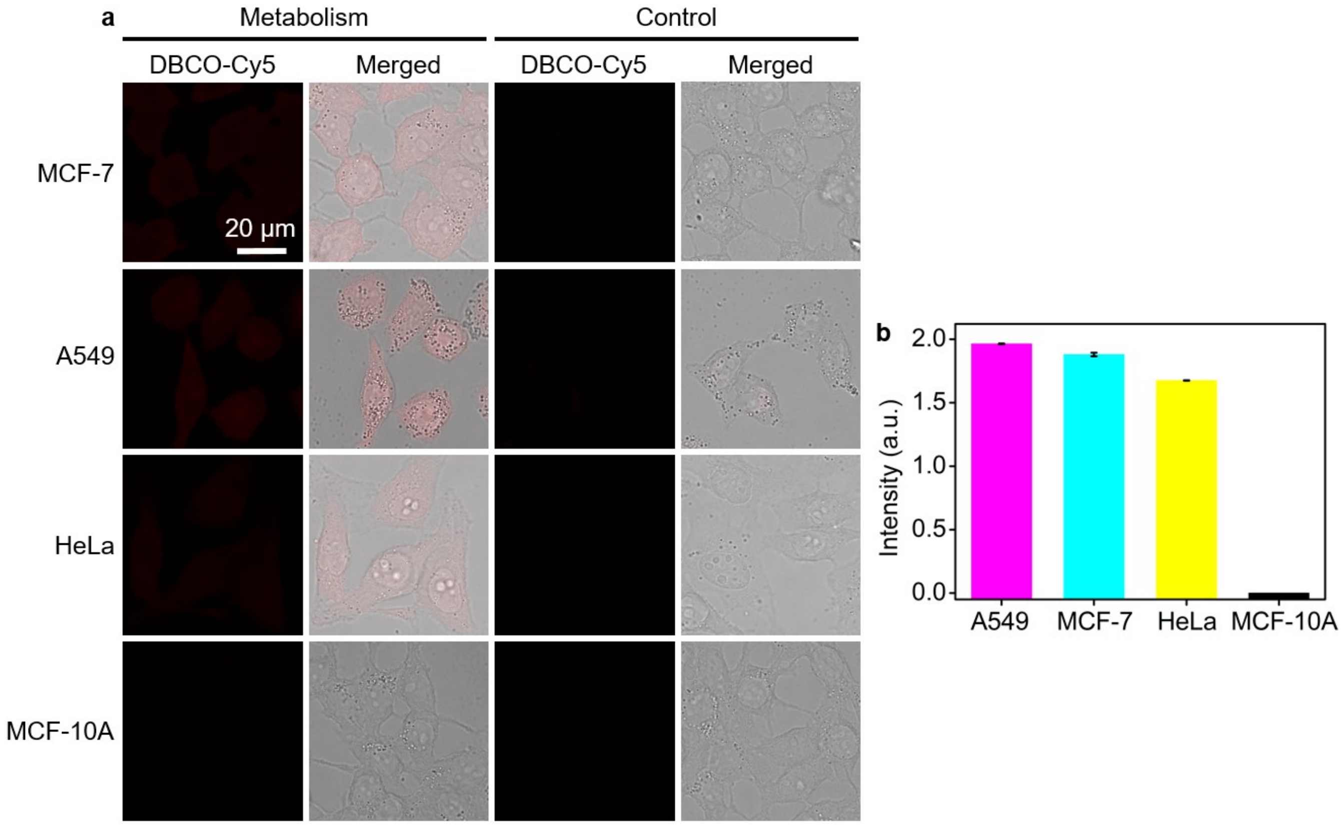

Figure S13 a CLSM images of OGCs in MCF-7, A549, HeLa and MCF-10A cells. **b** Statistic fluorescence intensities from (**a**). The data error bars indicate mean ± SD (n = 3).

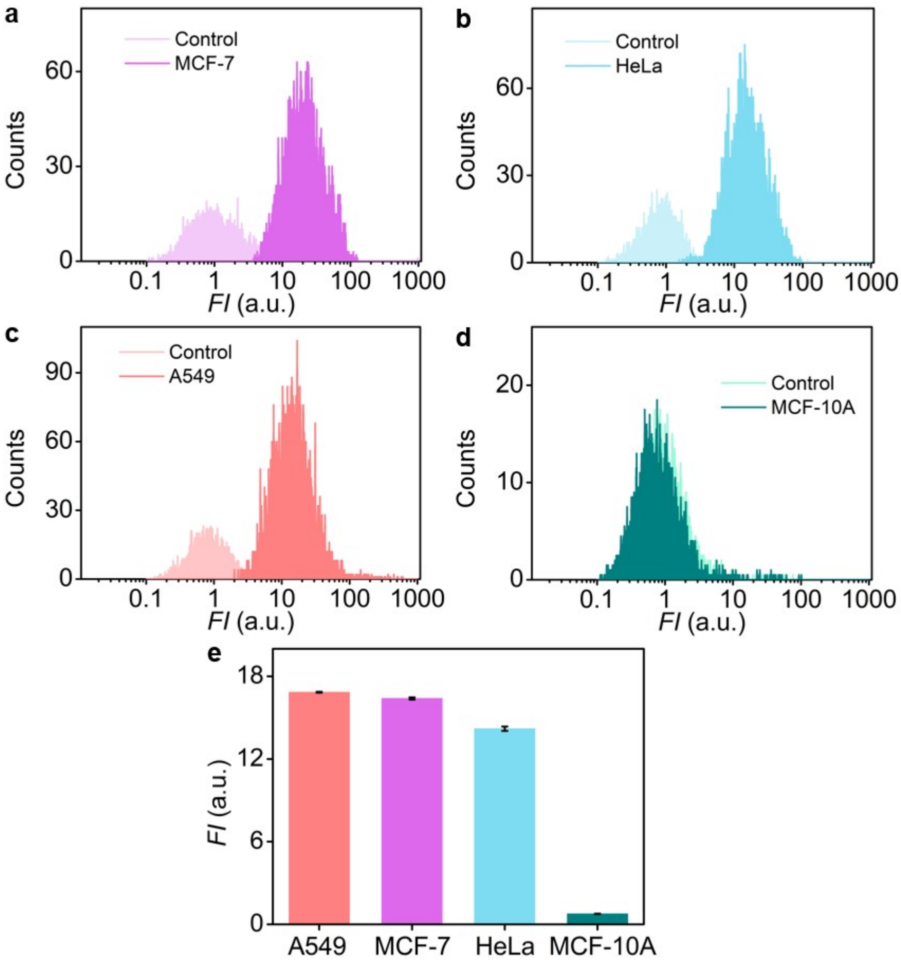

Figure S14 Flow cytometric analysis of O-GlcNAcylation in MCF-7 (**a**), HeLa (**b**), A549 (**c**) and MCF-10A (**d**) cells. **e** Statistic fluorescence intensities from (**a**) to (**d**). The data error bars indicate mean ± SD (n = 3).

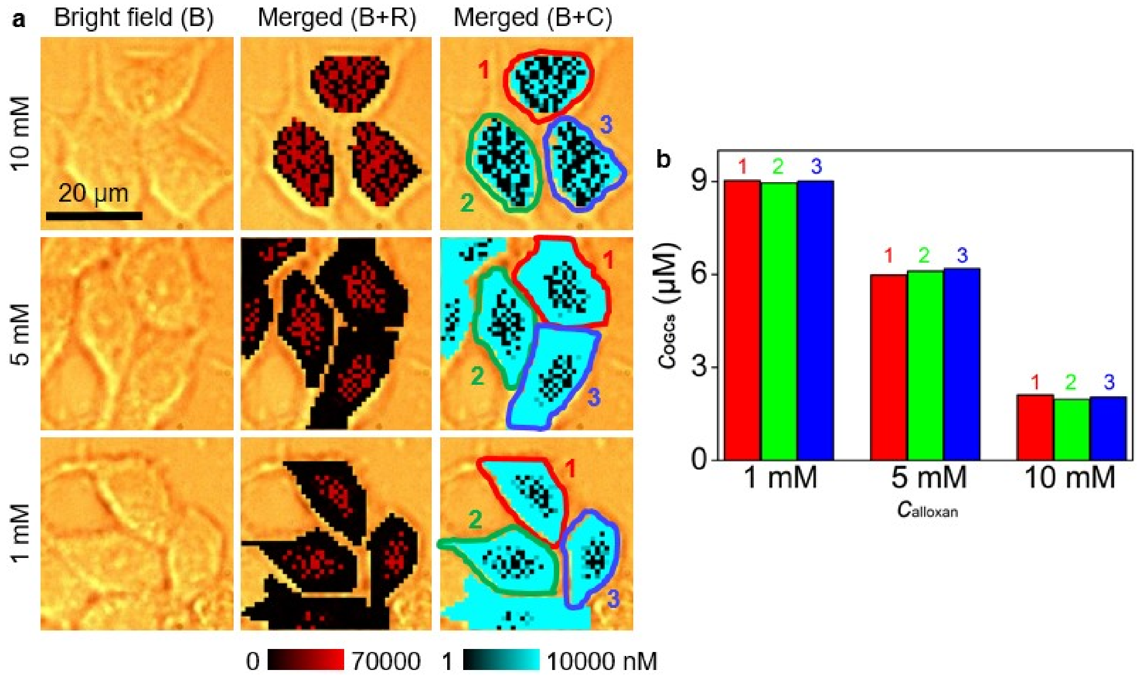

Figure S15 **a** SERS images (B+R) and O-GlcNAcylation mapping (B+C) of MCF-7 cells after inhibited with alloxan at different concentrations. **b** Statistical O-GlcNAcylation mapping intensities of every single cell in (**a**).

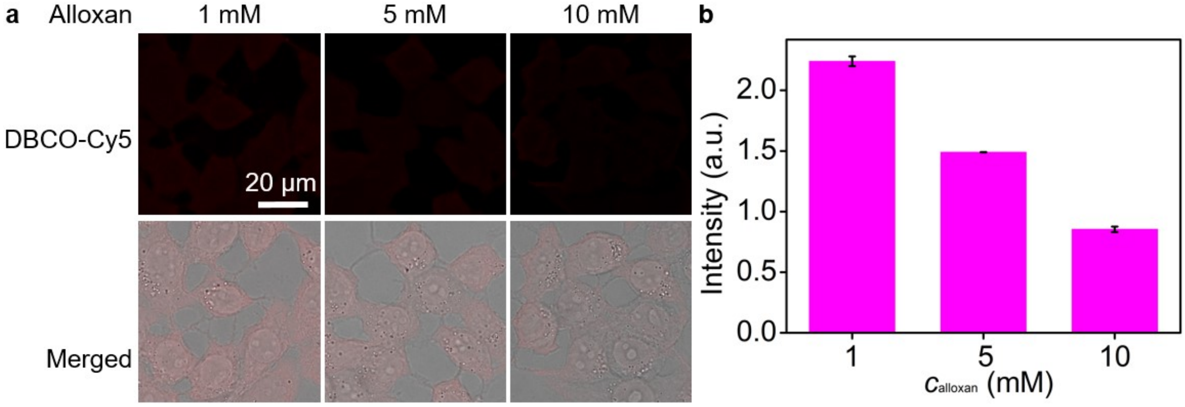

Figure S16 **a** CLSM images of MCF-7 cells treated with different concentrations of alloxan. **b** Statistic fluorescence intensities from (**a**). The data error bars indicate mean ± SD (n = 3).

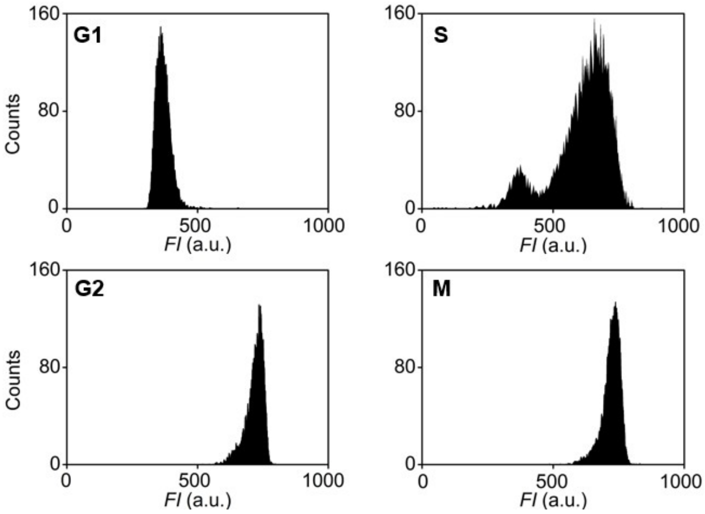

Figure S17 Flow cytometric analysis of MCF-7 cells synchronized at G1, S, G2, and M phases.

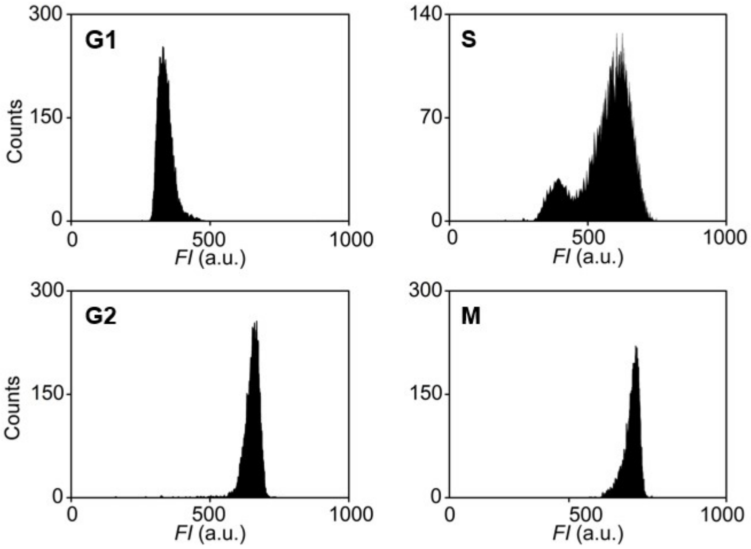

Figure S18 Flow cytometric analysis of GlcNAz metabolically labelled MCF-7 cells at G1, S, G2, and M phases.

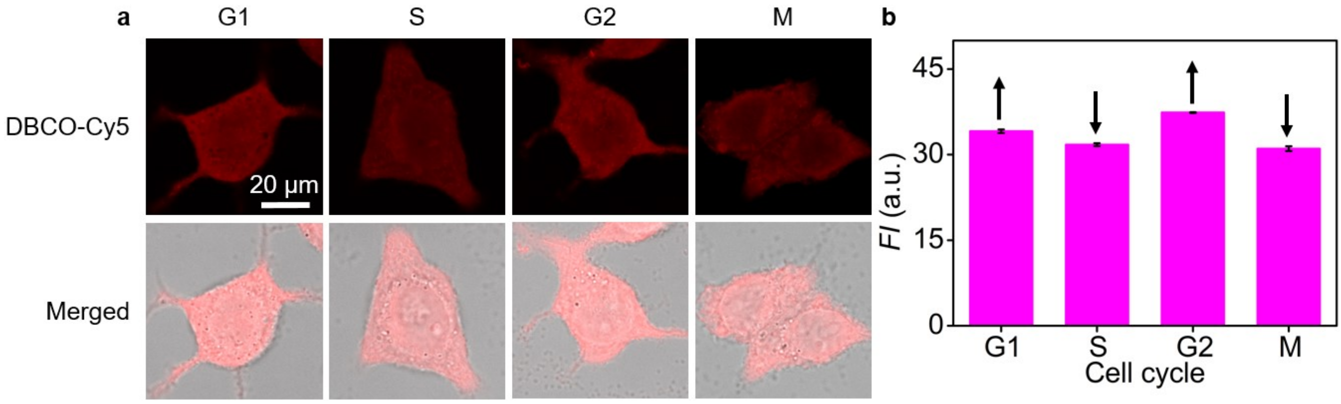

Figure S19 a CLSM images of OGCs in MCF-7 cells at G1, S, G2, and M phases. **b** Statistic fluorescence intensities from (**a**). The black arrows indicate the fluorescence changes. The data error bars indicate mean ± SD (n = 3).

References

1. G. Frens, *Nat. Phys. Sci.,* 1973, **241**, 20-22.

2. W. Haiss, N. T. K. Thanh, J. Aveyard, and D. G. Fernig, *Anal. Chem.,* 2007, **79**, 4215-4221.

3. Y. J. Yang, Y. L. Chen, J. X. Guo, H. P. Liu, and H. X. Ju, *iScience,* 2021, **24**, 102980.

4. R. V. Giles, D.G. Spiller, J. Grzybowski, R.E. Clark, P. Nicklin, and D.M. Tidd, *Nucleic Acids Res.,* 1998, **26**, 1567-1575.
